# Supplementary material for: Long-term Christmas Bird Counts describe neotropical urban bird diversity
Source: PLoS One. 2023 Feb 1;18(2):e0272754. doi: 10.1371/journal.pone.0272754 (PMC9891503; doi:10.1371/journal.pone.0272754)

**Legends of supporting information**

| ***Supporting material 1*** | ***S1 Table.*** *List of sites in each sampled circle (abbreviations used in the manuscript), total number of years counted, periods (years) of count, total number of observers (obs.), total kilometers surveyed (km), and duration of all observations (duration), for three cities with Christmas Bird Counts in Colombia.* |
| --- | --- |
| ***Supporting material 2*** | ***S2 Table.*** *Bird species observed in Bogotá, Cali, and Medellín between 2001 and 2018. Urban category: category of response to urbanization according to the definition proposed by Fischer et al. 2015, and based on expert criteria.* Data used for the manuscript is openly available at the National Audubon Society Christmas Bird count website ([https://netapp.audubon.org/cbcobservation/](https://netapp.audubon.org/cbcobservation/))). Data can also be obtain from each of the local count coordinators at:  Asociación Bogotana de Ornitología ABO: <https://www.avesbogota.org/> [abo@avesbogota.org](mailto:abo@avesbogota.org)  Asociación para el estudio y conservación de aves acuáticas en Colombia CALIDIRS <https://calidris.org.co/> [calidris@calidris.org.co](mailto:calidris@calidris.org.co)  Sociedad Antioqueña de Ornitología SAO: <https://sao.org.co/> [sao@une.net.co](mailto:sao@une.net.co) |
| ***Supporting material 3*** | ***S3 Table.*** *Accumulation curves for species/individual in each of three Colombian cities between 2001 and 2018 for the CBC.* |
| ***Supporting material 4*** | ***S4 Table.*** *Rarefaction curves of species / individuals by site within each city between 2001 and 2018 for the CBC in Colombia.* |
| ***Supporting material 5*** | ***S1 Figure.*** *Non-metric multidimensional scaling analysis (NMDS) based on abundances of the species reported between 2001 and 2018 in Bogotá CBC (all categories included).* |
| ***Supporting material 6*** | ***S2 Figure.*** *Non-metric multidimensional scaling analysis (NMDS) based on abundances of the species reported between 2001 and 2018 in Medellín CBC (for all categories included).* |
| ***Supporting material 7*** | ***S3 Figure.*** *Non-metric multidimensional scaling analysis (NMDS) based on abundances of the species reported between 2001 and 2018 in Cali CBC ( all categories included).* |

**S1 Table.** List of sites in each circle (abbreviations used in the manuscript), the total number of years counted, periods of count (Years), total number of observers (Obs.), total number of kilometers surveyed (km), and duration of all observations (hrs:minutes:seconds), for three cities with Christmas Bird Counts in Colombia. Urban area*; Periurban área **. For the Bogotá circle, site were not closer to one another more than 3.1km, while Cali sites were at least 803m apart, and Medellin sites more than 3.6km apart.

| **City** | **Number of Years** | **Years** | **Obs.** | **km** | **Duration** |
| --- | --- | --- | --- | --- | --- |
| **Bogotá, Sabana de Bogotá Christmas Bird Count Circle (ABO)** | | | | | |
| Aurora Alta (AUA)** | 18 | 2001-2018 | 89 | 136 | 136:15:44 |
| Humedal de Córdoba-Parque Niza (HCOR)* | 18 | 2001-2018 | 164 | 71,8 | 117:19:07 |
| Humedal de Guaymaral (HGU)** | 14 | 2001-2003, 2008-2018 | 72 | 46,1 | 52:59:10 |
| Humedal Jaboque (HJB)** | 17 | 2001-2004, 2006-2018 | 135 | 106,5 | 97:39:20 |
| Humedal Juan Amarillo (HJA)** | 8 | 2003-2007, 2013-2014, 2018 | 58 | 40,75 | 42:45:00 |
| Humedal la Conejera (HCON)** | 18 | 2001-2018 | 100 | 43,15 | 77:20:38 |
| Jardín Botánico (JBO)* | 16 | 2001, 2003-2016, 2018 | 145 | 36,6 | 64:17:09 |
| Parque La Florida (HPF)** | 16 | 2001-2003, 2006-2018 | 101 | 59,22 | 80:00:56 |
| Parque Simón Bolívar (PSB)* | 15 | 2001, 2003-2012, 2014-2018 | 100 | 49,08 | 54:17:09 |
| Santa María del Lago (HSL)* | 15 | 2001-2006, 2008-2013, 2016-2018 | 108 | 36,05 | 48:13:50 |
| Tabio (TBO)** | 18 | 2001-2018 | 90 | 91,8 | 119:19:30 |
| Valle de Teusacá (VTE)** | 16 | 2001-2016 | 78 | 122,1 | 25:50:46 |
| **Cali, Cordillera Occidental Christmas Bird Count (CCO)** | | | | | |
| Chicoral-Dapa (CHIDA)** | 11 | 2004, 2006-2010, 2014-2018 | 70 | 45 | 52:23:34 |
| Chicoral-Escuela (CHIES)** | 13 | 2004-2011, 2013-2014, 2016-2018 | 51 | 39 | 51:25:50 |
| Chicoral-Montebello (CHIMO)** | 14 | 2003-2012, 2014, 2016-2018 | 68 | 42,7 | 70:12:25 |
| Kilómetro 18 (KM18)** | 10 | 2003-2004, 2008, 2010-2013, 2016-2018 | 38 | 30,7 | 50:03:46 |
| **Medellín** | | | | | |
| **North Christmas Counts Bird Circle (SAO1)** | | | | | |
| Cerro el Volador (CV)* | 12 | 2001, 2003-2004, 2009-2012, 2014-2018 | 44 | 22,48 | 41:48:00 |
| Jardín Botánico de Medellín (JB)* | 13 | 2001-2002, 2004, 2008-2012, 2014-2018 | 31 | 22,2 | 33:40:00 |
| **South Christmas Counts Bird Circle (SAO2)** | | | | | |
| Alto de San Sebastián (ASSE)** | 10 | 2001, 2003, 2008-2011, 2014-2017 | 59 | 32,05 | 41:30:00 |
| Parque Ecológico La Romera (PER)** | 9 | 2001-2002, 2008-2009, 2011-2012, 2014-2016 | 39 | 30,9 | 46:46:00 |
| Parque Ecológico Recreativo Alto de San Miguel (PERASM)** | 8 | 2001, 2009-2011, 2014-2017 | 27 | 32,46 | 49:37:00 |

**S2 Table.** Identity and registered individuals of the bird species observed in Bogotá, Cali and Medellín between 2001 and 2018. Urban category = category of response to urbanization processes according to the definition proposed by Fischer et al. 2015 and based on the criteria of an expert. The taxonomy follows that proposed by Remsen et al. 2021 to August 2021. Feeding habit: CAR:Carnivore, INS:Insectivore, FRU:Frugivore, GRA:Granivore, NEC:Nectarivore, OMN:Omnivore. Urban category: AVO:Avoider, DWE:Dweller, UTI:Utilizer. Data used for the manuscript is openly available at the National Audubon Society Christmas Bird count website ([https://netapp.audubon.org/cbcobservation/](https://netapp.audubon.org/cbcobservation/))). Data can also be obtain from each of the local count coordinators at:

Asociación Bogotana de Ornitología ABO: <https://www.avesbogota.org/> [abo@avesbogota.org](mailto:abo@avesbogota.org)

Asociación para el estudio y conservación de aves acuáticas en Colombia CALIDIRS <https://calidris.org.co/> [calidris@calidris.org.co](mailto:calidris@calidris.org.co)

Sociedad Antioqueña de Ornitología SAO: <https://sao.org.co/> [sao@une.net.co](mailto:sao@une.net.co)

| **Taxonomy** | | **Feeding habit** | **Urban category** | **Status** | **Libro rojo de aves de Colombia** | **UICN** | **City** | | |
| --- | --- | --- | --- | --- | --- | --- | --- | --- | --- |
| **Family** | **Species** | **CAR, INS, FRU, GRA, NEC, OMN** | **AVO, DWE, UTI** | **Migrator, Resident** |  | **Red List of Threatened Species** | **Bogotá** | **Cali** | **Medellín** |
|  |  |  |  |  |  |  | **Individuals** | **Individuals** | **Individuals** |
| Tinamidae | *Tinamus tao* | INS | AVO | Resident | LC | VU |  | 1 |  |
| Tinamidae | *Crypturellus soui* | INS | AVO | Resident |  | LC |  | 8 | 1 |
| Anatidae | *Dendrocygna bicolor* | OMN | AVO | Resident |  | LC | 1 |  |  |
| Anatidae | *Dendrocygna autumnalis* | OMN | AVO | Resident |  | LC | 21 |  |  |
| Anatidae | *Anser anser* | OMN | AVO | Domestic |  | LC | 1 |  |  |
| Anatidae | *Cairina moschata* | OMN | UTI | Domestic |  | LC | 3 |  | 1 |
| Anatidae | *Spatula discors* | OMN | AVO | Migrant |  | LC | 5501 |  |  |
| Anatidae | *Anas platyrhynchos* | OMN | AVO | Resident |  | LC |  |  | 13 |
| Anatidae | *Anas andium* | OMN | AVO | Resident | LC | LC | 9 |  |  |
| Anatidae | *Anas flavirostris* | OMN | AVO | Resident |  | LC | 9 |  |  |
| Anatidae | *Aythya affinis* | OMN | AVO | Resident |  | LC | 26 |  |  |
| Anatidae | *Nomonyx dominicus* | GRA | AVO | Resident |  | LC | 2 |  |  |
| Anatidae | *Oxyura jamaicensis* | OMN | AVO | Resident | EN | LC | 1427 |  |  |
| Cracidae | *Chamaepetes goudotii* | FRU | AVO | Resident |  | LC |  | 13 | 26 |
| Cracidae | *Penelope montagnii* | FRU | AVO | Resident |  | LC | 50 |  |  |
| Cracidae | *Ortalis columbiana* | FRU | AVO | Resident |  | LC |  | 37 | 51 |
| Cracidae | *Ortalis guttata* | FRU | AVO | Resident |  | LC |  |  | 14 |
| Odontophoridae | *Colinus cristatus* | INS | AVO | Resident |  | LC | 6 |  |  |
| Odontophoridae | *Odontophorus hyperythrus* | GRA | AVO | Resident | LC | LC |  | 58 | 6 |
| Podicipedidae | *Tachybaptus dominicus* | OMN | AVO | Resident |  | LC | 1 |  |  |
| Podicipedidae | *Podilymbus podiceps* | OMN | AVO | Resident |  | LC | 393 |  |  |
| Columbidae | *Columba livia* | GRA | DWE | Resident |  | LC | 63 | 12 | 5 |
| Columbidae | *Patagioenas fasciata* | GRA | AVO | Resident |  | LC | 322 | 8 | 141 |
| Columbidae | *Patagioenas cayennensis* | GRA | AVO | Resident |  | LC |  | 17 |  |
| Columbidae | *Patagioenas subvinacea* | GRA | AVO | Resident |  | LC |  |  | 2 |
| Columbidae | *Geotrygon montana* | GRA | AVO | Resident |  | LC |  | 9 |  |
| Columbidae | *Leptotila verreauxi* | GRA | AVO | Resident |  | LC |  | 7 | 11 |
| Columbidae | *Zentrygon frenata* | GRA | AVO | Resident |  | LC |  | 4 |  |
| Columbidae | *Zenaida auriculata* | GRA | DWE | Resident |  | LC | 12754 | 51 | 450 |
| Columbidae | *Columbina talpacoti* | GRA | DWE | Resident |  | LC | 2 | 71 | 306 |
| Cuculidae | *Crotophaga major* | INS | UTI | Resident |  | LC | 36 |  |  |
| Cuculidae | *Crotophaga ani* | INS | UTI | Resident |  | LC | 1 | 31 | 65 |
| Cuculidae | *Tapera naevia* | INS | AVO | Resident |  | LC |  |  | 3 |
| Cuculidae | *Piaya cayana* | GRA | AVO | Resident |  | LC |  | 29 | 21 |
| Cuculidae | *Coccyzus americanus* | INS | UTI | Migrant |  | LC | 3 |  | 2 |
| Caprimulgidae | *Chordeiles minor* | INS | UTI | Migrant |  | LC | 1 |  | 1 |
| Caprimulgidae | *Systellura longirostris* | INS | UTI | Resident |  | LC | 85 |  | 1 |
| Apodidae | *Streptoprocne rutila* | INS | UTI | Resident |  | LC |  | 32 | 34 |
| Apodidae | *Streptoprocne zonaris* | INS | UTI | Resident |  | LC |  | 1090 | 510 |
| Trochilidae | *Florisuga mellivora* | NEC | UTI | Resident |  | LC |  | 71 |  |
| Trochilidae | *Phaethornis yaruqui* | NEC | AVO | Resident |  | LC |  | 1 |  |
| Trochilidae | *Phaethornis guy* | NEC | UTI | Resident |  | LC |  | 58 |  |
| Trochilidae | *Phaethornis syrmatophorus* | NEC | UTI | Resident |  | LC |  | 13 | 6 |
| Trochilidae | *Doryfera ludovicae* | NEC | AVO | Resident |  | LC |  | 1 |  |
| Trochilidae | *Schistes geoffroyi* | NEC | AVO | Resident |  | LC |  | 14 |  |
| Trochilidae | *Colibri delphinae* | NEC | AVO | Resident |  | LC |  | 27 |  |
| Trochilidae | *Colibri cyanotus* | NEC | DWE | Resident |  |  | 5 |  | 43 |
| Trochilidae | *Colibri coruscans* | NEC | DWE | Resident |  | LC | 1574 | 1 | 42 |
| Trochilidae | *Heliothryx barroti* | NEC | AVO | Resident |  | LC |  | 3 |  |
| Trochilidae | *Anthracothorax nigricollis* | NEC | AVO | Resident |  | LC |  | 3 | 35 |
| Trochilidae | *Heliangelus amethysticollis* | NEC | AVO | Resident |  | LC | 1 |  |  |
| Trochilidae | *Heliangelus exortis* | NEC | AVO | Resident |  | LC |  | 1 | 19 |
| Trochilidae | *Adelomyia melanogenys* | NEC | AVO | Resident |  | LC |  | 94 | 1 |
| Trochilidae | *Aglaiocercus kingii* | NEC | AVO | Resident |  | LC |  | 53 | 1 |
| Trochilidae | *Lesbia victoriae* | NEC | AVO | Resident |  | LC | 5 |  |  |
| Trochilidae | *Lesbia nuna* | NEC | UTI | Resident |  | LC | 109 |  |  |
| Trochilidae | *Ramphomicron microrhynchum* | NEC | AVO | Resident |  | LC | 2 |  |  |
| Trochilidae | *Metallura tyrianthina* | NEC | AVO | Resident |  | LC | 104 |  | 17 |
| Trochilidae | *Haplophaedia aureliae* | NEC | AVO | Resident |  | LC |  | 15 | 7 |
| Trochilidae | *Eriocnemis vestita* | NEC | AVO | Resident |  | LC | 210 |  |  |
| Trochilidae | *Eriocnemis cupreoventris* | NEC | AVO | Resident | LC | NT | 18 |  |  |
| Trochilidae | *Coeligena coeligena* | NEC | AVO | Resident |  | LC |  | 43 | 7 |
| Trochilidae | *Coeligena torquata* | NEC | AVO | Resident |  | LC |  | 1 | 25 |
| Trochilidae | *Coeligena bonapartei* | NEC | AVO | Resident |  | LC | 53 |  |  |
| Trochilidae | *Coeligena helianthea* | NEC | AVO | Resident |  | LC | 81 |  |  |
| Trochilidae | *Lafresnaya lafresnayi* | NEC | AVO | Resident |  | LC | 3 | 1 |  |
| Trochilidae | *Ensifera ensifera* | NEC | AVO | Resident |  | LC | 5 |  |  |
| Trochilidae | *Boissonneaua flavescens* | NEC | AVO | Resident |  | LC |  | 12 | 5 |
| Trochilidae | *Ocreatus underwoodii* | NEC | AVO | Resident |  | LC |  | 103 | 14 |
| Trochilidae | *Heliodoxa rubinoides* | NEC | AVO | Resident |  | LC |  | 38 | 1 |
| Trochilidae | *Heliomaster longirostris* | NEC | AVO | Resident |  | LC |  | 1 |  |
| Trochilidae | *Chaetocercus mulsant* | NEC | UTI | Resident |  | LC | 129 | 3 | 8 |
| Trochilidae | *Philodice mitchellii* | NEC | AVO | Resident |  | LC |  | 25 |  |
| Trochilidae | *Chlorostilbon melanorhynchus* | NEC | AVO | Resident |  | LC |  | 35 | 10 |
| Trochilidae | *Chlorostilbon mellisugus* | NEC | AVO | Resident |  | LC |  | 1 | 10 |
| Trochilidae | *Chalybura buffonii* | NEC | AVO | Resident |  | LC |  | 1 |  |
| Trochilidae | *Thalurania colombica* | NEC | AVO | Resident |  | LC |  | 14 |  |
| Trochilidae | *Saucerottia saucerottei* | NEC | AVO | Resident |  | LC |  | 12 | 25 |
| Trochilidae | *Amazilia tzacatl* | NEC | UTI | Resident |  | LC |  | 15 | 94 |
| Trochilidae | *Uranomitra franciae* | NEC | AVO | Resident |  | LC |  | 25 | 17 |
| Trochilidae | *Chrysuronia grayi* | NEC | AVO | Resident |  | LC |  | 1 |  |
| Rallidae | *Rallus semiplumbeus* | OMN | AVO | Resident | EN | VU | 187 |  |  |
| Rallidae | *Porphyrio martinica* | OMN | AVO | Local Migrant |  | LC | 71 |  | 1 |
| Rallidae | *Laterallus albigularis* | INS | AVO | Resident |  | LC |  |  | 12 |
| Rallidae | *Mustelirallus erythrops* | INS | AVO | Resident |  | LC | 1 |  |  |
| Rallidae | *Porphyriops melanops* | OMN | AVO | Resident | EN | LC | 679 |  |  |
| Rallidae | *Porzana carolina* | OMN | AVO | Resident |  | LC | 9 |  |  |
| Rallidae | *Gallinula galeata* | INS | AVO | Resident |  | LC | 4793 |  |  |
| Rallidae | *Fulica americana* | OMN | AVO | Resident |  | LC | 5194 |  |  |
| Heliornithidae | *Heliornis fulica* | OMN | AVO | Local Migrant |  | LC | 1 |  |  |
| Charadriidae | *Vanellus chilensis* | INS | UTI | Resident |  | LC | 711 | 44 | 43 |
| Scolopacidae | *Gallinago nobilis* | INS | AVO | Resident |  | NT | 36 |  |  |
| Scolopacidae | *Actitis macularius* | INS | AVO | Migrant |  | LC | 143 |  | 4 |
| Scolopacidae | *Tringa solitaria* | INS | AVO | Migrant |  | LC | 454 |  | 3 |
| Scolopacidae | *Tringa melanoleuca* | INS | AVO | Migrant |  | LC | 301 |  |  |
| Scolopacidae | *Tringa flavipes* | INS | AVO | Migrant |  | LC | 505 |  |  |
| Jacanidae | *Jacana jacana* | INS | AVO | Resident |  | LC | 3 |  |  |
| Laridae | *Leucophaeus atricilla* | CAR | AVO | Resident |  | LC | 1 |  |  |
| Laridae | *Phaetusa simplex* | CAR | AVO | Resident |  | LC | 2 |  |  |
| Phalacrocoracidae | *Phalacrocorax brasilianus* | CAR | AVO | Resident |  | LC | 96 |  | 13 |
| Ardeidae | *Nycticorax nycticorax* | OMN | AVO | Resident |  | LC | 149 |  | 17 |
| Ardeidae | *Butorides virescens* | INS | AVO | Resident |  | LC | 38 |  |  |
| Ardeidae | *Butorides striata* | INS | AVO | Resident |  | LC | 119 |  | 15 |
| Ardeidae | *Bubulcus ibis* | INS | UTI | Resident |  | LC | 5016 | 35 | 53 |
| Ardeidae | *Ardea herodias* | INS | AVO | Resident |  | LC | 7 |  |  |
| Ardeidae | *Ardea alba* | INS | AVO | Resident |  | LC | 253 |  | 5 |
| Ardeidae | *Egretta thula* | INS | UTI | Resident |  | LC | 4 | 1 | 8 |
| Ardeidae | *Egretta caerulea* | INS | AVO | Resident |  | LC | 27 |  |  |
| Threskiornithidae | *Phimosus infuscatus* | INS | AVO | Resident |  | LC | 2192 |  | 112 |
| Cathartidae | *Coragyps atratus* | CAR | DWE | Resident |  | LC | 2895 | 104 | 288 |
| Cathartidae | *Cathartes aura* | CAR | DWE | Resident |  | LC | 9 | 11 | 30 |
| Pandionidae | *Pandion haliaetus* | CAR | UTI | Migrant |  | LC | 16 |  | 2 |
| Accipitridae | *Elanus leucurus* | CAR | DWE | Resident |  | LC | 179 |  | 1 |
| Accipitridae | *Chondrohierax uncinatus* | CAR | AVO | Resident |  | LC | 1 |  |  |
| Accipitridae | *Elanoides forficatus* | CAR | AVO | Migrant |  | LC |  |  | 4 |
| Accipitridae | *Spizaetus ornatus* | CAR | AVO | Resident |  | NT |  | 4 |  |
| Accipitridae | *Ictinia plumbea* | CAR | AVO | Resident |  | LC | 1 |  |  |
| Accipitridae | *Accipiter striatus* | CAR | AVO | Resident |  | LC | 32 |  |  |
| Accipitridae | *Morphnarchus princeps* | CAR | AVO | Resident |  | LC |  | 1 |  |
| Accipitridae | *Rupornis magnirostris* | CAR | UTI | Resident |  | LC | 75 | 57 | 63 |
| Accipitridae | *Parabuteo leucorrhous* | CAR | AVO | Resident |  | LC | 2 |  | 1 |
| Accipitridae | *Geranoaetus albicaudatus* | CAR | AVO | Resident |  | LC |  |  | 2 |
| Accipitridae | *Geranoaetus polyosoma* | CAR | AVO | Resident |  | LC |  |  | 8 |
| Accipitridae | *Buteo platypterus* | CAR | UTI | Migrant |  | LC | 172 | 26 | 9 |
| Accipitridae | *Buteo swainsoni* | CAR | UTI | Migrant |  | LC | 5 |  |  |
| Tytonidae | *Tyto alba* | CAR | DWE | Resident |  | LC | 4 |  |  |
| Strigidae | *Megascops albogularis* | CAR | AVO | Resident |  | LC | 33 |  |  |
| Strigidae | *Megascops choliba* | CAR | AVO | Resident |  | LC | 16 |  |  |
| Strigidae | *Ciccaba albitarsis* | CAR | UTI | Resident |  | LC | 3 |  |  |
| Strigidae | *Glaucidium jardinii* | CAR | AVO | Resident |  | LC | 20 |  |  |
| Strigidae | *Asio clamator* | CAR | UTI | Resident |  | LC | 13 |  |  |
| Strigidae | *Asio stygius* | CAR | UTI | Resident |  | LC | 2 |  |  |
| Strigidae | *Asio flammeus* | CAR | UTI | Resident |  | LC | 2 |  |  |
| Trogonidae | *Pharomachrus auriceps* | FRU | AVO | Resident |  | LC |  | 35 |  |
| Trogonidae | *Pharomachrus antisianus* | FRU | AVO | Resident |  | LC |  | 3 |  |
| Trogonidae | *Trogon collaris* | FRU | AVO | Resident |  | LC |  | 32 | 1 |
| Trogonidae | *Trogon personatus* | FRU | AVO | Resident |  | LC |  | 8 | 5 |
| Momotidae | *Momotus momota* | INS | AVO | Resident |  | LC |  | 2 | 11 |
| Momotidae | *Momotus aequatorialis* | INS | AVO | Resident |  | LC |  | 143 | 40 |
| Alcedinidae | *Megaceryle torquata* | INS | AVO | Resident |  | LC |  | 1 | 7 |
| Alcedinidae | *Chloroceryle americana* | CAR | AVO | Resident |  | LC |  |  | 1 |
| Capitonidae | *Eubucco bourcierii* | INS | AVO | Resident |  | LC |  | 132 | 20 |
| Ramphastidae | *Aulacorhynchus albivitta* | OMN | AVO | Resident |  | LC |  | 30 | 46 |
| Ramphastidae | *Aulacorhynchus haematopygus* | OMN | AVO | Resident |  | LC |  | 133 |  |
| Ramphastidae | *Andigena nigrirostris* | OMN | AVO | Resident | NT | LC |  |  | 14 |
| Picidae | *Picumnus olivaceus* | INS | AVO | Resident |  | LC |  |  | 6 |
| Picidae | *Picumnus granadensis* | INS | AVO | Resident |  | LC |  | 9 |  |
| Picidae | *Melanerpes formicivorus* | INS | AVO | Resident |  | LC |  | 59 | 10 |
| Picidae | *Melanerpes rubricapillus* | INS | AVO | Resident |  | LC |  |  | 69 |
| Picidae | *Dryobates fumigatus* | INS | AVO | Resident |  | LC | 109 | 23 | 3 |
| Picidae | *Dryobates kirkii* | INS | AVO | Resident |  | LC |  |  | 1 |
| Picidae | *Dryobates dignus* | INS | AVO | Resident |  | LC |  | 5 |  |
| Picidae | *Campephilus pollens* | INS | AVO | Resident |  | LC |  |  | 3 |
| Picidae | *Dryocopus lineatus* | INS | AVO | Resident |  | LC |  | 3 | 6 |
| Picidae | *Colaptes rubiginosus* | INS | AVO | Resident |  | LC |  | 59 | 22 |
| Picidae | *Colaptes rivolii* | INS | AVO | Resident |  | LC | 29 |  | 5 |
| Picidae | *Colaptes punctigula* | INS | AVO | Resident |  | LC |  |  | 12 |
| Falconidae | *Caracara plancus* | CAR | UTI | Resident |  | LC |  |  | 1 |
| Falconidae | *Milvago chimachima* | CAR | UTI | Resident |  | LC |  | 6 | 11 |
| Falconidae | *Falco sparverius* | CAR | UTI | Resident |  | LC | 20 | 2 | 2 |
| Falconidae | *Falco columbarius* | CAR | UTI | Resident |  | LC | 14 |  |  |
| Falconidae | *Falco peregrinus* | CAR | UTI | Migrant |  | LC | 7 |  | 1 |
| Psittacidae | *Bolborhynchus lineola* | FRU | AVO | Resident |  | LC |  |  | 10 |
| Psittacidae | *Brotogeris jugularis* | FRU | UTI | Resident |  | LC |  |  | 123 |
| Psittacidae | *Pionus menstruus* | GRA | AVO | Resident |  | LC |  | 24 |  |
| Psittacidae | *Pionus chalcopterus* | GRA | AVO | Resident |  | LC |  | 12 |  |
| Psittacidae | *Amazona autumnalis* | GRA | UTI | Resident |  | LC |  | 5 | 6 |
| Psittacidae | *Amazona ochrocephala* | GRA | UTI | Resident |  | LC | 8 |  | 24 |
| Psittacidae | *Amazona amazonica* | FRU | AVO | Resident |  | LC |  |  | 38 |
| Psittacidae | *Amazona mercenarius* | GRA | AVO | Resident |  | LC |  | 51 |  |
| Psittacidae | *Forpus conspicillatus* | FRU | UTI | Resident |  | LC | 346 | 11 | 85 |
| Psittacidae | *Eupsittula pertinax* | FRU | AVO | Resident |  | LC |  |  | 11 |
| Psittacidae | *Ara severus* | FRU | UTI | Resident |  | LC |  |  | 22 |
| Psittacidae | *Ara macao* | FRU | UTI | Resident |  | LC |  |  | 6 |
| Psittacidae | *Psittacara wagleri* | GRA | AVO | Resident |  | NT |  | 520 |  |
| Thamnophilidae | *Thamnophilus doliatus* | INS | AVO | Resident |  | LC |  |  | 4 |
| Thamnophilidae | *Thamnophilus multistriatus* | INS | AVO | Resident |  | LC |  | 21 | 50 |
| Thamnophilidae | *Thamnophilus unicolor* | INS | AVO | Resident |  | LC |  | 24 |  |
| Thamnophilidae | *Dysithamnus mentalis* | INS | AVO | Resident |  | LC |  | 35 |  |
| Thamnophilidae | *Myrmotherula schisticolor* | INS | UTI | Resident |  | LC |  | 21 |  |
| Thamnophilidae | *Drymophila caudata* | INS | AVO | Resident |  | NT |  | 1 | 7 |
| Thamnophilidae | *Drymophila striaticeps* | INS | AVO | Resident |  | LC |  | 2 | 10 |
| Grallariidae | *Grallaria squamigera* | INS | AVO | Resident |  | LC | 37 |  |  |
| Grallariidae | *Grallaria guatimalensis* | INS | AVO | Resident |  | LC |  | 4 |  |
| Grallariidae | *Grallaria ruficapilla* | INS | AVO | Resident |  | LC | 264 |  | 42 |
| Grallariidae | *Grallaria rufocinerea* | INS | AVO | Resident |  | VU |  |  | 2 |
| Grallariidae | *Grallaria nuchalis* | INS | AVO | Resident |  | LC |  |  | 11 |
| Grallariidae | *Grallaria rufula* | INS | AVO | Resident |  | LC | 29 |  |  |
| Grallariidae | *Grallaricula nana* | INS | AVO | Resident |  | LC |  |  | 10 |
| Rhinocryptidae | *Acropternis orthonyx* | INS | AVO | Resident |  | LC |  |  | 2 |
| Rhinocryptidae | *Myornis senilis* | INS | AVO | Resident |  | LC |  |  | 2 |
| Rhinocryptidae | *Scytalopus latrans* | INS | AVO | Resident |  | LC |  |  | 26 |
| Rhinocryptidae | *Scytalopus vicinior* | INS | AVO | Resident |  | LC |  | 24 |  |
| Rhinocryptidae | *Scytalopus stilesi* | INS | AVO | Resident | EN | LC |  |  | 11 |
| Rhinocryptidae | *Scytalopus griseicollis* | INS | AVO | Resident |  | LC | 211 |  |  |
| Rhinocryptidae | *Scytalopus latebricola* | INS | AVO | Resident |  | NT |  | 1 |  |
| Rhinocryptidae | *Scytalopus spillmanni* | INS | AVO | Resident |  | LC |  |  | 1 |
| Furnariidae | *Sclerurus obscurior* | INS | AVO | Resident |  | LC |  | 2 |  |
| Furnariidae | *Dendrocincla tyrannina* | INS | AVO | Resident |  | LC |  | 1 |  |
| Furnariidae | *Glyphorynchus spirurus* | INS | AVO | Resident |  | LC |  | 1 |  |
| Furnariidae | *Dendrocolaptes picumnus* | INS | AVO | Resident |  | LC |  | 4 |  |
| Furnariidae | *Xiphocolaptes promeropirhynchus* | INS | AVO | Resident |  | LC | 4 | 2 |  |
| Furnariidae | *Xiphorhynchus lachrymosus* | INS | AVO | Resident |  | LC |  | 1 |  |
| Furnariidae | *Xiphorhynchus triangularis* | INS | AVO | Resident |  | LC |  | 6 | 1 |
| Furnariidae | *Lepidocolaptes souleyetii* | INS | AVO | Resident |  | LC |  | 7 |  |
| Furnariidae | *Lepidocolaptes lacrymiger* | INS | AVO | Resident |  | LC |  | 71 | 6 |
| Furnariidae | *Xenops minutus* | INS | AVO | Resident |  | LC |  | 16 |  |
| Furnariidae | *Xenops rutilans* | INS | AVO | Resident |  | LC |  | 25 | 1 |
| Furnariidae | *Premnornis guttuliger* | INS | AVO | Resident |  | LC |  | 2 | 4 |
| Furnariidae | *Anabacerthia striaticollis* | INS | UTI | Resident |  | LC |  | 28 |  |
| Furnariidae | *Syndactyla subalaris* | INS | AVO | Resident |  | LC |  | 27 |  |
| Furnariidae | *Dendroma rufa* | INS | AVO | Resident |  | LC |  | 4 |  |
| Furnariidae | *Thripadectes ignobilis* | INS | AVO | Resident |  | LC |  | 4 |  |
| Furnariidae | *Thripadectes holostictus* | INS | AVO | Resident |  | LC |  |  | 9 |
| Furnariidae | *Thripadectes virgaticeps* | INS | AVO | Resident |  | LC |  | 12 |  |
| Furnariidae | *Premnoplex brunnescens* | INS | AVO | Resident |  | LC |  | 12 | 6 |
| Furnariidae | *Margarornis squamiger* | INS | AVO | Resident |  | LC | 29 | 1 |  |
| Furnariidae | *Hellmayrea gularis* | INS | AVO | Resident |  | LC | 11 |  |  |
| Furnariidae | *Cranioleuca erythrops* | INS | AVO | Resident |  | LC |  | 118 |  |
| Furnariidae | *Synallaxis brachyura* | INS | AVO | Resident |  | LC |  | 15 |  |
| Furnariidae | *Synallaxis subpudica* | INS | AVO | Resident |  | LC | 824 |  |  |
| Furnariidae | *Synallaxis albescens* | INS | AVO | Resident |  | LC |  | 1 | 24 |
| Furnariidae | *Synallaxis azarae* | INS | AVO | Resident |  | LC |  | 155 | 188 |
| Furnariidae | *Synallaxis unirufa* | INS | AVO | Resident |  | LC |  | 2 |  |
| Pipridae | *Chloropipo flavicapilla* | FRU | AVO | Resident | VU | VU |  | 3 | 2 |
| Pipridae | *Masius chrysopterus* | INS | AVO | Resident |  | LC |  | 38 |  |
| Cotingidae | *Pipreola riefferii* | FRU | AVO | Resident |  | LC |  | 32 | 11 |
| Cotingidae | *Ampelion rubrocristatus* | FRU | AVO | Resident |  | LC | 90 |  |  |
| Cotingidae | *Ampelion rufaxilla* | FRU | AVO | Resident | VU | LC |  | 1 | 2 |
| Tityridae | *Pachyramphus versicolor* | FRU | AVO | Resident |  | LC |  | 32 | 1 |
| Tityridae | *Pachyramphus rufus* | FRU | AVO | Resident |  | LC |  | 1 |  |
| Tityridae | *Pachyramphus polychopterus* | FRU | AVO | Resident |  | LC |  | 7 | 1 |
| Tyrannidae | *Pseudotriccus ruficeps* | INS | AVO | Resident |  | LC |  |  | 1 |
| Tyrannidae | *Phylloscartes poecilotis* | INS | AVO | Resident |  | LC |  | 6 |  |
| Tyrannidae | *Phylloscartes ophthalmicus* | INS | AVO | Resident |  | LC |  | 22 | 4 |
| Tyrannidae | *Mionectes striaticollis* | INS | AVO | Resident |  | LC |  | 16 | 6 |
| Tyrannidae | *Mionectes olivaceus* | INS | AVO | Resident |  | LC |  | 6 | 1 |
| Tyrannidae | *Mionectes oleagineus* | INS | AVO | Resident |  | LC |  |  | 2 |
| Tyrannidae | *Leptopogon superciliaris* | INS | UTI | Resident |  | LC |  |  | 12 |
| Tyrannidae | *Leptopogon rufipectus* | INS | UTI | Resident |  | LC |  |  | 2 |
| Tyrannidae | *Tolmomyias sulphurescens* | INS | UTI | Resident |  | LC |  | 1 |  |
| Tyrannidae | *Lophotriccus pileatus* | INS | AVO | Resident |  | LC |  | 14 |  |
| Tyrannidae | *Hemitriccus granadensis* | INS | AVO | Resident |  | LC |  | 1 |  |
| Tyrannidae | *Poecilotriccus ruficeps* | INS | AVO | Resident |  | LC |  | 1 |  |
| Tyrannidae | *Todirostrum cinereum* | INS | AVO | Resident |  | LC | 1 | 10 | 51 |
| Tyrannidae | *Pyrrhomyias cinnamomeus* | INS | AVO | Resident |  | LC | 40 | 40 | 31 |
| Tyrannidae | *Zimmerius chrysops* | INS | UTI | Resident |  | LC |  | 176 | 57 |
| Tyrannidae | *Zimmerius viridiflavus* | INS | UTI | Resident |  | LC |  | 1 | 12 |
| Tyrannidae | *Camptostoma obsoletum* | INS | AVO | Resident |  | LC |  | 11 | 1 |
| Tyrannidae | *Elaenia flavogaster* | INS | UTI | Resident |  | LC | 25 | 9 | 40 |
| Tyrannidae | *Elaenia frantzii* | INS | UTI | Resident |  | LC | 276 | 3 | 37 |
| Tyrannidae | *Tyrannulus elatus* | INS | UTI | Resident |  | LC |  | 1 |  |
| Tyrannidae | *Phyllomyias griseiceps* | INS | AVO | Resident |  | LC |  | 3 |  |
| Tyrannidae | *Phyllomyias nigrocapillus* | INS | AVO | Resident |  | LC | 23 | 2 |  |
| Tyrannidae | *Phyllomyias cinereiceps* | INS | UTI | Resident |  | LC |  | 1 |  |
| Tyrannidae | *Phyllomyias uropygialis* | INS | AVO | Resident |  | LC | 7 |  |  |
| Tyrannidae | *Phyllomyias plumbeiceps* | INS | AVO | Resident |  | LC |  | 2 |  |
| Tyrannidae | *Phaeomyias murina* | INS | UTI | Resident |  | LC |  | 5 | 4 |
| Tyrannidae | *Mecocerculus leucophrys* | INS | UTI | Resident |  | LC | 655 |  | 6 |
| Tyrannidae | *Pseudocolopteryx acutipennis* | INS | AVO | Resident | CR | LC | 4 |  |  |
| Tyrannidae | *Serpophaga cinerea* | INS | AVO | Resident |  | LC | 72 |  | 50 |
| Tyrannidae | *Uromyias agilis* | INS | AVO | Resident |  | LC | 2 |  |  |
| Tyrannidae | *Attila spadiceus* | INS | AVO | Resident |  | LC |  | 61 |  |
| Tyrannidae | *Pitangus sulphuratus* | INS | UTI | Resident |  | LC | 23 | 15 | 218 |
| Tyrannidae | *Machetornis rixosa* | INS | UTI | Resident |  | LC |  |  | 10 |
| Tyrannidae | *Myiodynastes chrysocephalus* | INS | AVO | Resident |  | LC |  | 83 | 10 |
| Tyrannidae | *Myiodynastes luteiventris* | INS | UTI | Resident |  | LC | 1 |  | 4 |
| Tyrannidae | *Myiodynastes maculatus* | INS | AVO | Resident |  | LC |  |  | 11 |
| Tyrannidae | *Myiozetetes cayanensis* | INS | AVO | Resident |  | LC | 3 | 9 | 92 |
| Tyrannidae | *Myiozetetes similis* | INS | UTI | Resident |  | LC |  |  | 1 |
| Tyrannidae | *Tyrannus melancholicus* | INS | DWE | Resident |  | LC | 994 | 61 | 200 |
| Tyrannidae | *Tyrannus savana* | INS | UTI | Migrant |  | LC | 1 |  |  |
| Tyrannidae | *Tyrannus tyrannus* | INS | UTI | Migrant |  | LC | 37 |  | 1 |
| Tyrannidae | *Myiarchus tuberculifer* | INS | UTI | Resident |  | LC |  | 1 |  |
| Tyrannidae | *Myiarchus apicalis* | INS | UTI | Resident |  | LC |  | 3 |  |
| Tyrannidae | *Myiarchus cephalotes* | INS | AVO | Resident |  | LC |  | 1 | 24 |
| Tyrannidae | *Myiarchus crinitus* | INS | UTI | Resident |  | LC | 6 |  | 2 |
| Tyrannidae | *Myiophobus flavicans* | INS | AVO | Resident |  | LC |  | 1 |  |
| Tyrannidae | *Myiophobus fasciatus* | INS | AVO | Resident |  | LC |  |  | 7 |
| Tyrannidae | *Ochthoeca diadema* | INS | AVO | Resident |  | LC | 11 |  | 10 |
| Tyrannidae | *Ochthoeca cinnamomeiventris* | INS | AVO | Resident |  | LC |  |  | 2 |
| Tyrannidae | *Ochthoeca rufipectoralis* | INS | AVO | Resident |  | LC | 22 |  |  |
| Tyrannidae | *Ochthoeca fumicolor* | INS | AVO | Resident |  | LC | 3 |  | 3 |
| Tyrannidae | *Pyrocephalus rubinus* | INS | UTI | Resident |  | LC | 134 | 3 | 181 |
| Tyrannidae | *Myiotheretes striaticollis* | INS | AVO | Resident |  | LC | 2 |  | 2 |
| Tyrannidae | *Sayornis nigricans* | INS | UTI | Resident |  | LC | 11 | 3 | 54 |
| Tyrannidae | *Empidonax virescens* | INS | UTI | Migrant |  | LC |  | 7 | 1 |
| Tyrannidae | *Empidonax traillii* | INS | UTI | Migrant |  | LC |  | 1 |  |
| Tyrannidae | *Empidonax (alnorum-traillii)* | INS | UTI | Migrant |  | LC | 28 |  |  |
| Tyrannidae | *Contopus cooperi* | INS | UTI | Migrant | NT | NT | 11 |  |  |
| Tyrannidae | *Contopus fumigatus* | INS | UTI | Resident |  | LC | 10 | 10 | 3 |
| Tyrannidae | *Contopus sordidulus* | INS | UTI | Migrant |  | LC |  | 2 |  |
| Tyrannidae | *Contopus virens* | INS | UTI | Migrant |  | LC |  |  | 4 |
| Tyrannidae | *Contopus (virens-sordidulus)* | INS | UTI | Migrant |  | LC | 76 |  |  |
| Vireonidae | *Cyclarhis gujanensis* | INS | UTI | Resident |  | LC |  | 2 |  |
| Vireonidae | *Cyclarhis nigrirostris* | FRU | AVO | Resident |  | LC |  | 49 | 29 |
| Vireonidae | *Pachysylvia semibrunnea* | INS | AVO | Resident |  | LC |  | 14 | 3 |
| Vireonidae | *Vireo flavifrons* | INS | UTI | Resident |  | LC |  |  | 2 |
| Vireonidae | *Vireo leucophrys* | INS | UTI | Migrant |  | LC |  | 27 | 21 |
| Vireonidae | *Vireo olivaceus* | INS | UTI | Migrant |  | LC | 67 | 5 | 8 |
| Vireonidae | *Vireo flavoviridis* | INS | UTI | Migrant |  | LC | 8 |  |  |
| Corvidae | *Cyanolyca armillata* | INS | AVO | Resident |  | LC |  |  | 7 |
| Corvidae | *Cyanolyca viridicyanus* | OMN | AVO | Resident |  | NT |  |  | 1 |
| Corvidae | *Cyanocorax affinis* | INS | AVO | Resident |  | LC | 1 |  |  |
| Corvidae | *Cyanocorax yncas* | INS | AVO | Resident |  | LC |  | 7 | 54 |
| Hirundinidae | *Pygochelidon cyanoleuca* | INS | DWE | Migrant |  | LC |  | 202 | 412 |
| Hirundinidae | *Orochelidon murina* | INS | DWE | Resident |  | LC | 6003 | 6 |  |
| Hirundinidae | *Stelgidopteryx ruficollis* | INS | UTI | Resident |  | LC |  | 7 | 44 |
| Hirundinidae | *Riparia riparia* | INS | UTI | Migrant |  | LC | 9 |  |  |
| Hirundinidae | *Hirundo rustica* | INS | UTI | Migrant |  | LC | 19 | 4 |  |
| Hirundinidae | *Petrochelidon pyrrhonota* | INS | UTI | Migrant |  | LC | 2 |  |  |
| Troglodytidae | *Troglodytes aedon* | INS | DWE | Resident |  | LC | 1274 | 214 | 182 |
| Troglodytidae | *Troglodytes solstitialis* | INS | AVO | Resident |  | LC | 6 | 4 |  |
| Troglodytidae | *Cistothorus apolinari* | INS | AVO | Resident | CR | EN | 46 |  |  |
| Troglodytidae | *Pheugopedius mystacalis* | INS | AVO | Resident |  | LC |  | 38 | 26 |
| Troglodytidae | *Pheugopedius genibarbis* | INS | AVO | Resident |  | LC |  | 6 |  |
| Troglodytidae | *Cinnycerthia unirufa* | INS | AVO | Resident |  | LC | 177 |  |  |
| Troglodytidae | *Cinnycerthia olivascens* | INS | AVO | Resident |  | LC |  |  | 10 |
| Troglodytidae | *Cinnycerthia peruana* | INS | AVO | Resident |  | LC |  |  | 4 |
| Troglodytidae | *Henicorhina leucophrys* | INS | AVO | Resident |  | LC | 50 | 370 | 135 |
| Troglodytidae | *Cyphorhinus thoracicus* | INS | AVO | Resident | LC | LC |  | 54 |  |
| Polioptilidae | *Ramphocaenus melanurus* | INS | AVO | Resident |  | LC |  | 1 |  |
| Cinclidae | *Cinclus leucocephalus* | INS | AVO | Resident |  | LC |  |  | 3 |
| Turdidae | *Myadestes ralloides* | INS | AVO | Resident |  | LC |  | 203 | 77 |
| Turdidae | *Catharus aurantiirostris* | INS | UTI | Migrant |  | LC |  | 4 | 13 |
| Turdidae | *Catharus ustulatus* | INS | UTI | Migrant |  | LC | 91 | 53 | 31 |
| Turdidae | *Turdus leucops* | FRU | UTI | Resident |  | LC |  | 1 |  |
| Turdidae | *Turdus leucomelas* | FRU | AVO | Resident |  | LC | 1 |  |  |
| Turdidae | *Turdus obsoletus* | INS | AVO | Resident |  | LC |  | 1 |  |
| Turdidae | *Turdus ignobilis* | FRU | AVO | Resident |  | LC | 29 | 236 | 245 |
| Turdidae | *Turdus fuscater* | FRU | DWE | Resident |  | LC | 5183 | 3 | 134 |
| Turdidae | *Turdus serranus* | FRU | AVO | Resident |  | LC |  | 9 |  |
| Mimidae | *Mimus gilvus* | INS | DWE | Resident |  | LC | 24 |  |  |
| Fringillidae | *Spinus spinescens* | GRA | DWE | Resident |  | LC | 797 | 12 | 1 |
| Fringillidae | *Spinus xanthogastrus* | GRA | UTI | Resident |  | LC |  | 18 | 8 |
| Fringillidae | *Spinus psaltria* | GRA | DWE | Resident |  | LC | 623 | 54 | 48 |
| Fringillidae | *Chlorophonia cyanocephala* | FRU | AVO | Resident |  | LC |  | 54 | 1 |
| Fringillidae | *Chlorophonia cyanea* | FRU | AVO | Resident |  | LC |  | 40 |  |
| Fringillidae | *Chlorophonia pyrrhophrys* | FRU | AVO | Resident |  | LC |  | 3 |  |
| Fringillidae | *Euphonia laniirostris* | FRU | UTI | Resident |  | LC |  | 7 | 20 |
| Fringillidae | *Euphonia xanthogaster* | FRU | UTI | Resident |  | LC |  | 139 | 1 |
| Passerellidae | *Chlorospingus flavopectus* | FRU | AVO | Resident |  | LC |  | 1 | 90 |
| Passerellidae | *Chlorospingus canigularis* | FRU | AVO | Resident |  | LC |  | 69 |  |
| Passerellidae | *Chlorospingus semifuscus* | FRU | AVO | Resident |  | LC |  | 1 |  |
| Passerellidae | *Arremon assimilis* | FRU | AVO | Resident |  | LC | 41 |  |  |
| Passerellidae | *Arremon torquatus* | FRU | AVO | Resident |  | LC | 41 |  |  |
| Passerellidae | *Arremon brunneinucha* | FRU | AVO | Resident |  | LC |  | 50 | 19 |
| Passerellidae | *Zonotrichia capensis* | GRA | DWE | Resident |  | LC | 6587 | 485 | 181 |
| Passerellidae | *Atlapetes albinucha* | FRU | AVO | Resident |  | LC |  | 82 | 60 |
| Passerellidae | *Atlapetes schistaceus* | FRU | AVO | Resident |  | LC | 115 |  |  |
| Passerellidae | *Atlapetes pallidinucha* | FRU | AVO | Resident |  | LC | 124 |  |  |
| Passerellidae | *Atlapetes latinuchus* | FRU | AVO | Resident |  | LC |  | 1 | 9 |
| Passerellidae | *Atlapetes rufinucha* | FRU | AVO | Resident |  | LC |  |  | 15 |
| Icteridae | *Sturnella magna* | INS | AVO | Resident |  | NT | 290 |  |  |
| Icteridae | *Amblycercus holosericeus* | FRU | AVO | Resident |  | LC | 16 |  |  |
| Icteridae | *Psarocolius angustifrons* | OMN | AVO | Resident |  | LC |  |  | 18 |
| Icteridae | *Cacicus chrysonotus* | FRU | AVO | Resident |  | LC | 15 |  | 4 |
| Icteridae | *Cacicus cela* | FRU | AVO | Resident |  | LC | 8 |  |  |
| Icteridae | *Icterus icterus* | FRU | UTI | Resident | VU | LC | 29 |  |  |
| Icteridae | *Icterus chrysater* | FRU | UTI | Resident |  | LC | 281 | 14 |  |
| Icteridae | *Icterus galbula* | FRU | UTI | Resident |  | LC |  | 1 | 4 |
| Icteridae | *Icterus nigrogularis* | FRU | UTI | Resident |  | LC | 97 |  |  |
| Icteridae | *Molothrus oryzivorus* | OMN | UTI | Resident |  | LC | 8 |  | 1 |
| Icteridae | *Molothrus bonariensis* | OMN | UTI | Resident |  | LC | 2260 | 85 | 98 |
| Icteridae | *Quiscalus lugubris* | INS | UTI | Resident |  | LC | 108 |  |  |
| Icteridae | *Quiscalus mexicanus* | INS | AVO | Resident |  | LC |  |  | 2 |
| Icteridae | *Hypopyrrhus pyrohypogaster* | FRU | AVO | Resident | VU | VU |  |  | 241 |
| Icteridae | *Gymnomystax mexicanus* | INS | UTI | Resident |  | LC | 4 |  |  |
| Icteridae | *Chrysomus icterocephalus* | INS | AVO | Resident |  | LC | 2072 |  |  |
| Parulidae | *Parkesia noveboracensis* | INS | UTI | Migrant |  | LC | 136 | 1 | 30 |
| Parulidae | *Vermivora chrysoptera* | INS | AVO | Migrant | LC | NT |  | 3 |  |
| Parulidae | *Mniotilta varia* | INS | UTI | Migrant |  | LC | 28 | 47 | 36 |
| Parulidae | *Protonotaria citrea* | INS | UTI | Migrant |  | LC | 6 |  | 21 |
| Parulidae | *Leiothlypis peregrina* | INS | UTI | Migrant |  | LC | 229 |  | 29 |
| Parulidae | *Geothlypis philadelphia* | INS | UTI | Migrant |  | LC | 14 | 19 | 22 |
| Parulidae | *Setophaga ruticilla* | INS | UTI | Migrant |  | LC | 61 | 2 | 7 |
| Parulidae | *Setophaga cerulea* | INS | UTI | Migrant | VU | NT | 1 | 2 |  |
| Parulidae | *Setophaga pitiayumi* | INS | AVO | Migrant |  | LC |  | 56 |  |
| Parulidae | *Setophaga castanea* | INS | UTI | Migrant |  | LC | 2 | 1 | 23 |
| Parulidae | *Setophaga fusca* | INS | UTI | Migrant |  | LC | 332 | 547 | 163 |
| Parulidae | *Setophaga petechia* | INS | UTI | Migrant |  | LC | 127 |  | 58 |
| Parulidae | *Setophaga striata* | INS | UTI | Migrant |  | NT | 23 | 1 |  |
| Parulidae | *Myiothlypis luteoviridis* | INS | AVO | Resident |  | LC |  |  | 4 |
| Parulidae | *Myiothlypis nigrocristata* | INS | AVO | Resident |  | LC | 259 |  | 10 |
| Parulidae | *Myiothlypis coronata* | INS | AVO | Resident |  | LC | 1 | 42 | 66 |
| Parulidae | *Basileuterus rufifrons* | INS | AVO | Resident |  | LC |  |  | 10 |
| Parulidae | *Basileuterus culicivorus* | INS | AVO | Resident |  | LC |  | 11 | 1 |
| Parulidae | *Basileuterus tristriatus* | NEC | AVO | Resident |  | LC |  | 174 | 55 |
| Parulidae | *Cardellina canadensis* | INS | UTI | Migrant |  | LC | 11 | 102 | 23 |
| Parulidae | *Myioborus miniatus* | INS | AVO | Resident |  | LC |  | 347 | 113 |
| Parulidae | *Myioborus ornatus* | INS | AVO | Resident |  | LC | 84 |  | 52 |
| Cardinalidae | *Piranga flava* | FRU | UTI | Resident |  | LC |  | 4 | 3 |
| Cardinalidae | *Piranga rubra* | FRU | UTI | Migrant |  | LC | 203 | 98 | 124 |
| Cardinalidae | *Piranga olivacea* | FRU | UTI | Migrant |  | LC | 61 |  | 3 |
| Cardinalidae | *Pheucticus aureoventris* | FRU | AVO | Resident |  | LC | 73 |  |  |
| Cardinalidae | *Pheucticus ludovicianus* | FRU | UTI | Migrant |  | LC | 76 | 12 | 30 |
| Cardinalidae | *Passerina cyanea* | FRU | AVO | Resident |  | LC |  |  | 5 |
| Thraupidae | *Sericossypha albocristata* | FRU | AVO | Resident |  | VU |  |  | 2 |
| Thraupidae | *Catamblyrhynchus diadema* | INS | AVO | Resident |  | LC | 28 |  | 4 |
| Thraupidae | *Chlorophanes spiza* | FRU | AVO | Resident |  | LC |  | 60 |  |
| Thraupidae | *Hemithraupis guira* | FRU | AVO | Resident |  | LC |  | 1 |  |
| Thraupidae | *Conirostrum sitticolor* | INS | AVO | Resident |  | LC | 60 |  |  |
| Thraupidae | *Conirostrum rufum* | INS | AVO | Resident |  | LC | 293 |  |  |
| Thraupidae | *Sicalis flaveola* | GRA | DWE | Resident |  | LC | 75 | 20 | 150 |
| Thraupidae | *Sicalis luteola* | GRA | DWE | Resident |  | LC | 292 |  |  |
| Thraupidae | *Catamenia analis* | GRA | AVO | Resident |  | LC | 97 |  |  |
| Thraupidae | *Catamenia homochroa* | GRA | AVO | Resident |  | LC | 17 |  | 1 |
| Thraupidae | *Diglossa lafresnayii* | NEC | DWE | Resident |  | LC | 9 |  | 1 |
| Thraupidae | *Diglossa humeralis* | NEC | DWE | Resident |  | LC | 944 | 1 | 1 |
| Thraupidae | *Diglossa brunneiventris* | FRU | AVO | Resident |  | LC |  |  | 4 |
| Thraupidae | *Diglossa albilatera* | NEC | DWE | Resident |  | LC | 71 | 8 | 37 |
| Thraupidae | *Diglossa sittoides* | NEC | UTI | Resident |  | LC | 112 | 40 |  |
| Thraupidae | *Diglossa caerulescens* | NEC | UTI | Resident |  | LC | 136 |  | 16 |
| Thraupidae | *Diglossa cyanea* | NEC | UTI | Resident |  | LC | 99 | 6 | 31 |
| Thraupidae | *Haplospiza rustica* | GRA | UTI | Resident |  | LC |  | 2 | 4 |
| Thraupidae | *Volatinia jacarina* | GRA | UTI | Resident |  | LC |  | 20 | 13 |
| Thraupidae | *Creurgops verticalis* | INS | AVO | Resident | VU | LC |  | 3 |  |
| Thraupidae | *Tachyphonus rufus* | FRU | UTI | Resident |  | LC |  | 5 | 1 |
| Thraupidae | *Ramphocelus dimidiatus* | FRU | AVO | Resident |  | LC | 44 |  | 3 |
| Thraupidae | *Ramphocelus flammigerus* | FRU | AVO | Resident |  | LC |  | 244 | 45 |
| Thraupidae | *Cyanerpes cyaneus* | FRU | UTI | Resident |  | LC |  | 1 |  |
| Thraupidae | *Dacnis cayana* | FRU | AVO | Resident |  | LC |  | 4 |  |
| Thraupidae | *Sporophila minuta* | GRA | UTI | Resident |  | LC |  |  | 57 |
| Thraupidae | *Sporophila crassirostris* | GRA | AVO | Resident |  | LC |  |  | 4 |
| Thraupidae | *Sporophila intermedia* | GRA | UTI | Resident |  | LC |  | 2 | 2 |
| Thraupidae | *Sporophila luctuosa* | GRA | UTI | Resident |  | LC | 5 |  |  |
| Thraupidae | *Sporophila nigricollis* | GRA | UTI | Resident |  | LC |  | 195 | 76 |
| Thraupidae | *Sporophila schistacea* | GRA | UTI | Resident |  | LC |  | 9 | 5 |
| Thraupidae | *Saltator maximus* | FRU | UTI | Resident |  | LC |  | 2 |  |
| Thraupidae | *Saltator atripennis* | FRU | UTI | Resident |  | LC |  | 141 | 29 |
| Thraupidae | *Saltator coerulescens* | FRU | UTI | Resident |  | LC |  |  | 38 |
| Thraupidae | *Saltator striatipectus* | FRU | AVO | Resident |  | LC | 2 | 27 | 33 |
| Thraupidae | *Emberizoides herbicola* | GRA | AVO | Resident |  | LC |  |  | 1 |
| Thraupidae | *Pseudospingus verticalis* | FRU | AVO | Resident |  | LC | 107 |  |  |
| Thraupidae | *Cnemoscopus rubrirostris* | FRU | AVO | Resident |  | LC |  |  | 15 |
| Thraupidae | *Kleinothraupis atropileus* | FRU | AVO | Resident |  | LC |  |  | 7 |
| Thraupidae | *Sphenopsis frontalis* | FRU | AVO | Resident |  | LC |  | 19 | 9 |
| Thraupidae | *Sphenopsis melanotis* | FRU | AVO | Resident |  | LC | 8 |  |  |
| Thraupidae | *Thlypopsis superciliaris* | FRU | AVO | Resident |  | LC | 177 |  | 2 |
| Thraupidae | *Coereba flaveola* | FRU | UTI | Resident |  | LC |  | 49 | 250 |
| Thraupidae | *Tiaris olivaceus* | GRA | AVO | Resident |  | LC |  | 59 | 99 |
| Thraupidae | *Chlorochrysa nitidissima* | FRU | AVO | Resident | VU | NT |  | 14 |  |
| Thraupidae | *Iridosornis porphyrocephalus* | FRU | AVO | Resident | LC | NT |  | 4 |  |
| Thraupidae | *Pipraeidea melanonota* | FRU | AVO | Resident |  | LC | 2 | 8 | 1 |
| Thraupidae | *Dubusia taeniata* | FRU | AVO | Resident |  | LC | 24 |  |  |
| Thraupidae | *Anisognathus lacrymosus* | FRU | AVO | Resident |  | LC |  |  | 16 |
| Thraupidae | *Anisognathus igniventris* | FRU | AVO | Resident |  | LC | 415 |  |  |
| Thraupidae | *Anisognathus somptuosus* | FRU | AVO | Resident |  | LC |  | 110 | 33 |
| Thraupidae | *Buthraupis montana* | FRU | AVO | Resident |  | LC | 1 |  | 5 |
| Thraupidae | *Sporathraupis cyanocephala* | FRU | AVO | Resident |  | LC | 19 | 6 | 20 |
| Thraupidae | *Chlorornis riefferii* | FRU | AVO | Resident |  | LC |  |  | 10 |
| Thraupidae | *Cnemathraupis eximia* | FRU | AVO | Resident |  | LC | 13 |  |  |
| Thraupidae | *Chalcothraupis ruficervix* | FRU | AVO | Resident |  | LC |  | 122 | 1 |
| Thraupidae | *Stilpnia cyanicollis* | FRU | AVO | Resident |  |  |  | 35 | 11 |
| Thraupidae | *Stilpnia vitriolina* | FRU | AVO | Resident |  | LC | 17 | 148 | 153 |
| Thraupidae | *Stilpnia heinei* | FRU | AVO | Resident |  | LC |  | 52 | 111 |
| Thraupidae | *Tangara vassorii* | FRU | AVO | Resident |  | LC | 4 | 1 | 35 |
| Thraupidae | *Tangara nigroviridis* | FRU | AVO | Resident |  | LC |  | 101 | 30 |
| Thraupidae | *Tangara labradorides* | FRU | AVO | Resident |  | LC |  | 150 | 33 |
| Thraupidae | *Tangara gyrola* | FRU | UTI | Resident |  | LC |  | 16 | 20 |
| Thraupidae | *Tangara xanthocephala* | FRU | AVO | Resident |  | LC |  | 111 |  |
| Thraupidae | *Tangara arthus* | FRU | AVO | Resident |  | LC |  | 333 | 90 |
| Thraupidae | *Thraupis episcopus* | FRU | UTI | Resident |  | LC | 207 | 110 | 344 |
| Thraupidae | *Thraupis palmarum* | FRU | UTI | Resident |  | LC | 161 | 35 | 181 |

**S3 Table.** Results of the species/individual accumulation curves in each city between 2001 and 2018

| **City** | **Sp Obs** | **Sp Est** | **Ind** | **Chao 2** | **Chao 2 95% Low-Bou** | **Chao 2 95% CI Upp- Bou** | **Bootstrap** | **Rarefaction** | **Singletons** | **Doubletons** |
| --- | --- | --- | --- | --- | --- | --- | --- | --- | --- | --- |
| **Accumulation of species / individuals** | | | | | | | | | | |
| Bogotá, Sabana de Bogotá Christmas Bird Count Circle (ABO) | 214 | 214 | 83710 | 239,68 | 224,43 | 277,24 | 227,07 | 214 | 20 | 14 |
| Cali, Cordillera Occidental Christmas Bird Count (CCO) | 258 | 258 | 11490 | 308,99 | 284,45 | 356,3 | 283,29 | 258 | 47 | 16 |
| Medellín, Christmas Bird Count Circle(SAO1-2) | 270 | 270 | 10142 | 309,37 | 290,81 | 345,35 | 297,01 | 271 | 39 | 20 |
| **Accumulation of species / party-hours** | | | | | | | | | | |
| Bogotá, Sabana de Bogotá Christmas Bird Count Circle (ABO) | 214 | 214 | 97244 | 235,82 | 222,29 | 269,09 | 226,27 | 212 | 17 | 14 |
| Cali, Cordillera Occidental Christmas Bird Count (CCO) | 258 | 258 | 13438 | 306,28 | 282,44 | 352,45 | 281,94 | 254,6 | 37 | 21 |
| Medellín, Christmas Bird Count Circle(SAO1-2) | 270 | 270 | 16499 | 295,21 | 279,62 | 326,35 | 288,42 | 262,2 | 24 | 15 |

**S4 Table.** Results of the rarefaction curves of species / individuals by site in each city between 2001 and 2018.

| **Localidad** | **Sp Ob** | **Sp Est** | **Ind** | **Chao 2** | **Chao 2 95% CI Low-Bo** | **Chao 2 95% CI Up-Bou** | **Bootstrap** | **Rarefaction** | **Singletons** | **Doubletons** |
| --- | --- | --- | --- | --- | --- | --- | --- | --- | --- | --- |
| **Bogotá, Sabana de Bogotá Christmas Bird Count Circle (ABO)** | | | | | | | | | | |
| Aurora Alta (AUA)** | 106 | 106 | 4940 | 118 | 109,89 | 143,31 | 114 | 106 | 13 | 9 |
| Humedal de Córdoba-Parque Niza(HCOR)* | 109 | 109 | 9752 | 136 | 119,01 | 180,94 | 120 | 109 | 15 | 13 |
| Humedal de Guaymaral (HGU)** | 88 | 88 | 4686 | 106 | 94,53 | 136,79 | 98,35 | 88 | 13 | 9 |
| Humedal Jaboque (HJB)** | 87 | 87 | 13473 | 133,28 | 104,23 | 211,33 | 98,44 | 87 | 14 | 8 |
| Humedal Juan Amarillo (HJA)** | 74 | 74 | 4327 | 92,23 | 80,87 | 122,39 | 84,35 | 74 | 14 | 8 |
| Humedal la Conejera (HCON)** | 100 | 99 | 10451 | 121,86 | 107,07 | 163,7 | 108,54 | 98 | 17 | 8 |
| Jardín Botánico (JBO)* | 88 | 88 | 7030 | 130,19 | 104,52 | 195,72 | 100,11 | 88 | 22 | 8 |
| Parque La Florida (HPF)** | 91 | 91 | 9172 | 136,94 | 108,1 | 214,44 | 102,16 | 91 | 13 | 12 |
| Parque Simón Bolívar (PSB)* | 83 | 83 | 5795 | 100,3 | 89,57 | 128,58 | 94,25 | 83 | 15 | 11 |
| Santa María del Lago (HSL)* | 59 | 59 | 4820 | 73,99 | 63,73 | 106,45 | 66,27 | 59 | 11 | 8 |
| Tabio (TBO)** | 92 | 92 | 4935 | 121,75 | 101,94 | 181 | 100,85 | 92 | 16 | 6 |
| Valle de Teusacá (VTE)** | 55 | 55 | 4329 | 72,58 | 60,1 | 115,62 | 61,29 | 55 | 12 | 3 |
| **Cali, Cordillera Occidental Christmas Bird Count (CCO)** | | | | | | | | | | |
| Chicoral-Dapa (CHIDA)** | 190 | 190 | 2984 | 252,06 | 222,77 | 307,52 | 216,58 | 190 | 38 | 30 |
| Chicoral-Escuela (CHIES)** | 164 | 164 | 2668 | 190,26 | 176,19 | 220,56 | 183,08 | 164 | 28 | 23 |
| Chicoral-Montebello (CHIMO)** | 175 | 175 | 3370 | 210,77 | 191,71 | 251,55 | 193,54 | 173,04 | 26 | 20 |
| Kilómetro 18 (KM18)** | 190 | 190 | 2468 | 232,62 | 211,15 | 275,89 | 211,59 | 190 | 36 | 21 |
| **Medellín** | | | | | | | | | | |
| **North Christmas Counts Bird Circle (SAO1)** | | | | | | | | | | |
| Cerro el Volador (CV)* | 94 | 94 | 2112 | 129,81 | 106,88 | 193,57 | 104,03 | 94 | 18 | 7 |
| Jardín Botánico de Medellín (JB)* | 83 | 83 | 2070 | 111,36 | 93,71 | 158,11 | 93,7 | 83 | 18 | 5 |
| **South Christmas Counts Bird Circle (SAO2)** | | | | | | | | | | |
| Alto de San Sebastián (ASSE)** | 132 | 132 | 1736 | 192,19 | 161,63 | 254,28 | 153,11 | 132 | 37 | 7 |
| Parque Ecológico La Romera (PER)** | 144 | 144 | 2498 | 166,4 | 153,75 | 195,46 | 159,37 | 144 | 23 | 17 |
| Parque Ecológico Recreativo Alto de San Miguel (PERASM)** | 144 | 144 | 1726 | 193,29 | 168,31 | 243,94 | 164,72 | 144 | 35 | 18 |

**S5 Table. Estimated richness and abundance of birds by site in the three cities evaluated during the 18 years of the Christmas Bird Count in Colombia. Different letters indicate significant differences on the 5% level.**

|  | **Community** | | **Urbanization tolerance** | | | | | | **Feeding habit** | | | | | | | | | | | |
| --- | --- | --- | --- | --- | --- | --- | --- | --- | --- | --- | --- | --- | --- | --- | --- | --- | --- | --- | --- | --- |
|  | **Total** | | **Avoiders** | | **Utilizers** | | **Dwellers** | | **Insectivorous** | | **Frugivorous** | | **Nectarivorous** | | **Granivorous** | | **Carnivorous** | | **Omnivorous** | |
|  | **Rich** | **Abun** | **Rich** | **Abun** | **Rich** | **Abun** | **Rich** | **Abun** | **Rich** | **Abun** | **Rich** | **Abun** | **Rich** | **Abun** | **Rich** | **Abun** | **Rich** | **Abun** | **Rich** | **Abun** |
| **Bogotá** | | | | | | | | | | | | | | | | | | | | |
| TBO | 40.1(ef) | 274.2(a) | 19(e) | 74.8(c) | 9.7(bc) | 53.7(cd) | 11.3(b) | 145.6(b) | 15.3(ce) | 134.4(e) | 8.6(d) | 45.2(e) | 7(d) | 24.2(fg) | 5.3(b) | 54.6(fg) | 3.6(c) | 15.1(bc) | 0.3(a) | 0.6(a) |
| AUA | 51.5(g) | 274.4(a) | 33.7(f) | 156.7(e) | 8.6(bc) | 49.2(cd) | 9.3(ab) | 68.5(a) | 23.5(f) | 117.1(d) | 10.6(d) | 66.5(f) | 9.8(d) | 40.7(i) | 3.7(ab) | 31.8(i) | 3.9(a) | 18.4 (c) | 0.05(a) | 0.05(a) |
| VTE | 22.3(a) | 270.6(a) | 10.9(b) | 91.3(d) | 2.9 (a) | 10.1(a) | 8.4(ab) | 169.2(c) | 10.7(ab) | 128(de) | 1.2(a) | 6.6(a) | 0.8(a) | 1(a) | 2.8(a) | 43.2(a) | 1.8(b) | 18.1(c) | 5.1(de) | 72.9(d) |
| HCOR | 46.7(fg) | 541.7(e) | 14.9(be) | 260.9(g) | 21(d) | 78.3 (e) | 10.7(ab) | 202.5(ef) | 23.2(df) | 169.9(f) | 8.2(d) | 59.9 (f) | 3.2(bc) | 28(gh) | 3.6(ab) | 93(gh) | 3.8(ef) | 14.4(bc) | 4.7(ce) | 176.5(h) |
| HGU | 31.2(bc) | 334.6(b) | 11.6(bc) | 101.3(d) | 8.6(bc) | 48(c) | 11.1(b) | 185.8(d) | 13(bcd) | 97.4(c) | 2.7(ac) | 29(d) | 2.6(bc) | 11.1(d) | 4.4(ab) | 80.9(d) | 3.5(d) | 38.4(e) | 4.8(ce) | 77.8(d) |
| HJA | 31(bcd) | 442.3(d) | 11.9(bd) | 148.9(e) | 8.9(bc) | 55.9(cd) | 9.9(ab) | 237.6(g) | 14.4(be) | 131(de) | 3.3(bc) | 19.7(c) | 1.6(ac) | 13.6 (de) | 3.5 (ab) | 112.4(de) | 2.8(gh) | 64.3(f) | 5.1(ce) | 101.1(ef) |
| HCON | 38.5(de) | 580 (f) | 16.8 (de) | 218.9(f) | 10.8(bc) | 143.4(g) | 10.9(b) | 216.5(fg) | 18.7(ef) | 283.8(h) | 3.9(c) | 28.4 (d) | 2.3(bc) | 20.6 (f) | 3.9 (ab) | 99.1 (f) | 3.2 (fg) | 29.7(d) | 6.6(e) | 117.3(g) |
| HPF | 34 (ce) | 533.8 (e) | 15.3(cde) | 282.6(h) | 8.4(bc) | 88.1 (e) | 10.3(ab) | 163.2(c) | 16.7(de) | 207.3(g) | 1.9(ab) | 17.6 (c) | 1.6 (ab) | 3.7(b) | 4.2(ab) | 83.7(b) | 2.6(de) | 12.2(b) | 6.9(e) | 209.3(i) |
| HSL | 20.7(a) | 321.3(b) | 5.7 (a) | 92.3 (d) | 7.7(b) | 37.4 (b) | 7.3(a) | 191(de) | 7.7(a) | 41.9(a) | 3.7(bc) | 33.1(d) | 1.9(ac) | 22.8(fg) | 2.7(a) | 127.4(fg) | 0.9(h) | 3.1(a) | 3.9(cd) | 93(e) |
| HJB | 30.2 (bc) | 792.5(g) | 11 (b) | 347.1 (i) | 7.9(b) | 126.9 (f) | 11.3(b) | 318.5(h) | 14.8(ce) | 424.6(i) | 1.8(ab) | 13.1(b) | 1.6(ab) | 6.5(c) | 4.3(ab) | 214.2(c) | 2.9(j) | 26.4(d) | 4.9(de) | 107.7(fg) |
| JBO | 31.5(bc) | 380.3(c) | 3.5(a) | 8.3(a) | 17.6(d) | 56.7 (d) | 10.4(ab) | 315.4(h) | 11.9(bc) | 39.1(a) | 8.3(d) | 107.1(h) | 3.8(c) | 32.3(h) | 4.1(ab) | 188.5(h) | 2.2(i) | 4.7(a) | 1.3(ab) | 8.6(b) |
| PSB | 25.9(ab) | 413.9(d) | 4.8 (a) | 23.2 (b) | 12(c) | 45.7 (c) | 9.1(ab) | 344.9(i) | 11.6(bc) | 53.7(b) | 4(c) | 81.5(g) | 2.1(ac) | 19(ef) | 3.6(ab) | 231(ef) | 2(j) | 4.7(a) | 2.6(bc) | 23.9(c) |
| **Cali** | | | | | | | | | | | | | | | | | | | | |
| CHIDA | 57.5(a) | 165.6(a) | 34.7(a) | 85.5 (a) | 15.6(a) | 59.8(ab) | 7.2(a) | 20.4(a) | 25.7(ab) | 89.5(a) | 18.5(ab) | 44.1(a) | 5.3(a) | 9.6(a) | 5.4(a) | 17.1(a) | 1.3(a) | 1.9(a) | 1.3(a) | 3.4(a) |
| CHIES | 55.5(a) | 205.2(b) | 29.5(a) | 98.6(b) | 17(a) | 61.8(b) | 9.1(a) | 44.8(c) | 22.8(a) | 81.5(a) | 16.2(a) | 55.4(b) | 5.8(a) | 14.5(b) | 7.5(a) | 47.5(c) | 1.5(a) | 2.5(a) | 1.7(a) | 6.8(a) |
| CHIMO | 67.5(b) | 240.7(c) | 43.7(b) | 135.1(d) | 15.8(a) | 70(c) | 8(a) | 35.6(b) | 29.4(bc) | 118.1(b) | 19.1(ab) | 59 (b) | 10.1(b) | 23.1(c) | 6.1(a) | 31.6 (b) | 1.6 (a) | 5.9 (b) | 1.3 (a) | 3(a) |
| KM18 | 79.7(c) | 200.6(b) | 49.5(b) | 121.6(c) | 19.9(a) | 50.4(a) | 10.2(a) | 29.3(b) | 33.4(c) | 83.5(a) | 22.9(b) | 55.1(b) | 10.9(b) | 17.6(b) | 8.3(a) | 35.9(b) | 2.3(a) | 3.4(ab) | 1.9(a) | 5.1(ab) |
| **Medellín** | | | | | | | | | | | | | | | | | | | | |
| PERASM | 57.9(c) | 215.8(d) | 26.4(b) | 81.6(b) | 18.1(c) | 61.1(c) | 13.4(b) | 73(b) | 27.8(b) | 110.1(d) | 17(bc) | 63.9(c) | 3.8(bc) | 6(b) | 5.4(bc) | 23.3(b) | 2.9(a) | 8.6(bc) | 1.3(a) | 4.1(a) |
| PER | 67(c) | 245.7(e) | 35.5(c) | 112.8(c) | 17.9(bc) | 60.5(c) | 13.6(b) | 72.3(b) | 29.4(b) | 101.8(d) | 20.1(c) | 77.7(d) | 6.4(c) | 9.3(b) | 7.1(c) | 42.3(d) | 2.3(a) | 9(bc) | 1.6(a) | 5.2(a) |
| ASSE | 41.4(b) | 131.6 (a) | 31.5(bc) | 81(b) | 3(a) | 26.9(a) | 6.9(a) | 23.8(a) | 17.8(a) | 74.4(c) | 12.1(ab) | 29.8(a) | 6.3(c) | 15.6(c) | 2.6(a) | 6.5(a) | 1.9(a) | 3(a) | 0.7(a) | 2.4(a) |
| CV | 40.4(b) | 176 (c) | 6.9(a) | 18.7(a) | 21.5(c) | 79.8(d) | 12(b) | 77.5(b) | 17.8(a) | 61.8(b) | 10.1 (a) | 51.3(b) | 2.1(ab) | 6.3(b) | 6.9(c) | 39.4 (cd) | 2.8(a) | 12.5(c) | 0.8(a) | 4.7(a) |
| JB | 30.8(a) | 148.5(b) | 7.3(a) | 16.5(a) | 13.2(b) | 44.2(b) | 10.3(ab) | 87.8(c) | 13.5(a) | 50.5(a) | 9.2(a) | 51.3(b) | 1.5(a) | 3.1(a) | 3.2 (ab) | 33 (c) | 2.8(a) | 7.5(b) | 0.8(a) | 2.7(a) |

**S1 Fig.**  Non-metric multidimensional scaling analysis (NMDS) based on abundances of the species reported between 2001 and 2018 in Bogotá Christmas Count, for all analysis categories.


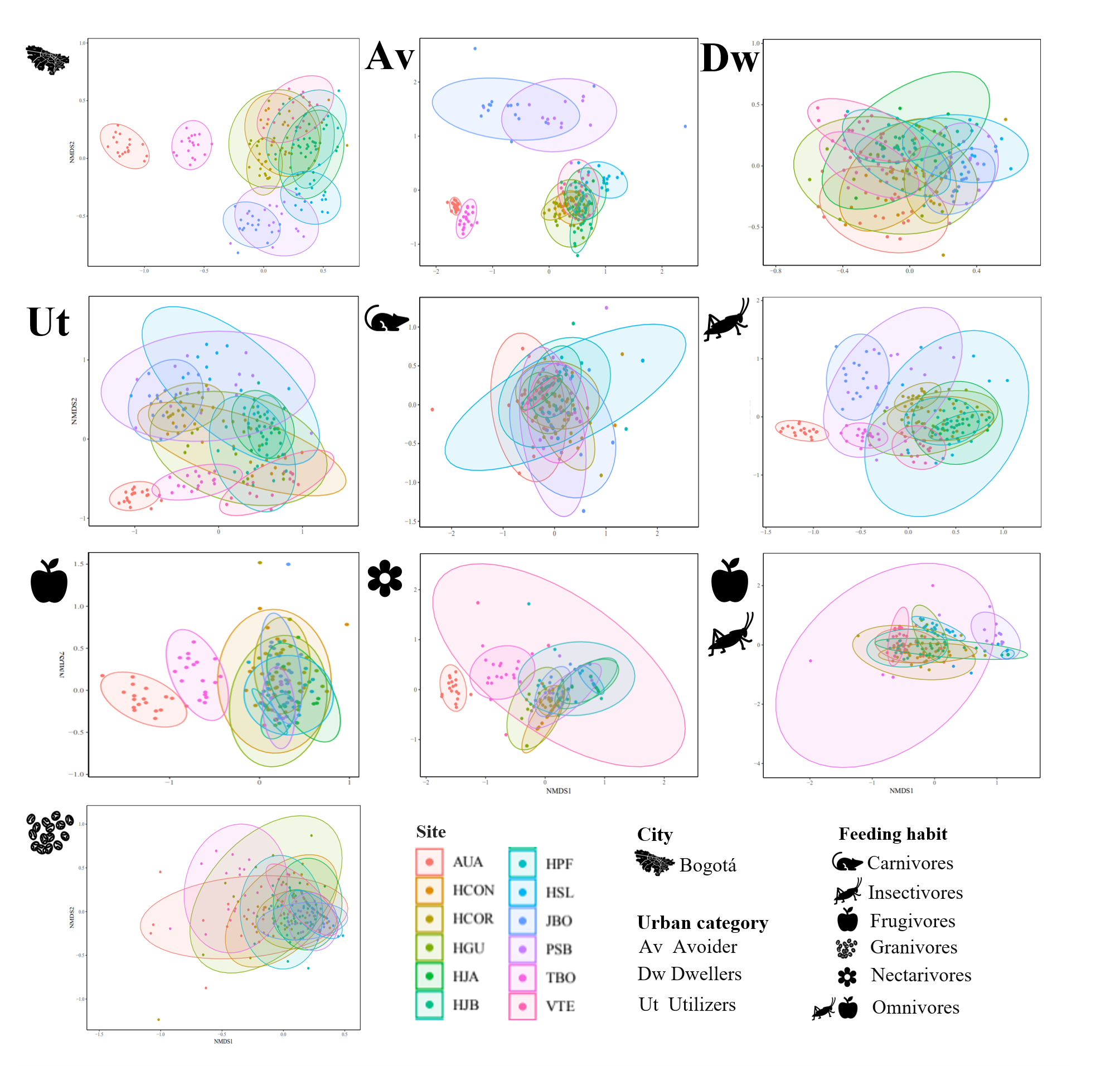


**S2 Fig.** Non-metric multidimensional scaling analysis (NMDS) based on abundances of the species reported between 2001 and 2018 in Medellín Christmas Count, for all analysis categories.


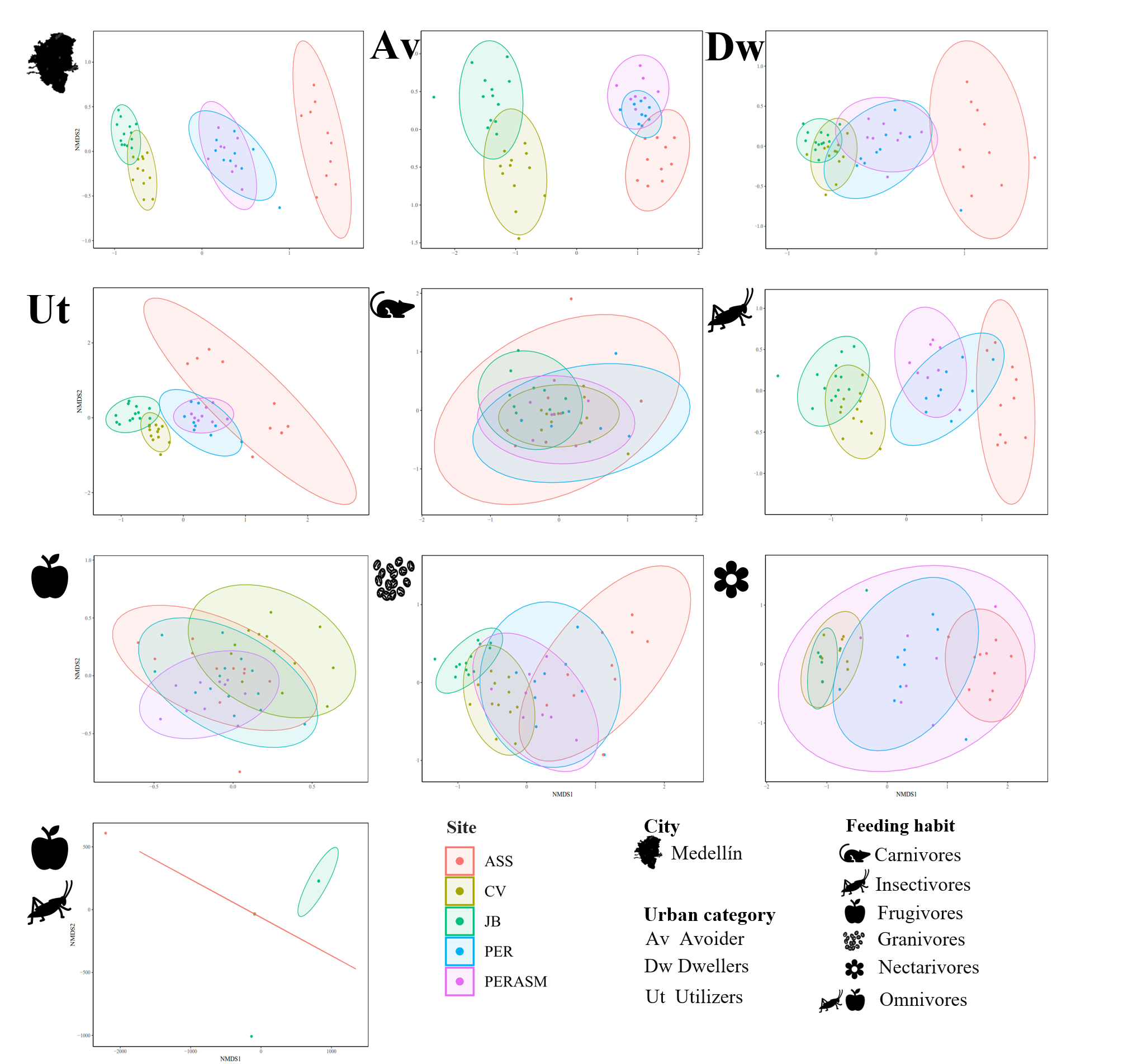


**S3 Fig.** Non-metric multidimensional scaling analysis (NMDS) based on abundances of the species reported between 2001 and 2018 in Cali Christmas Count, for all analysis categories.


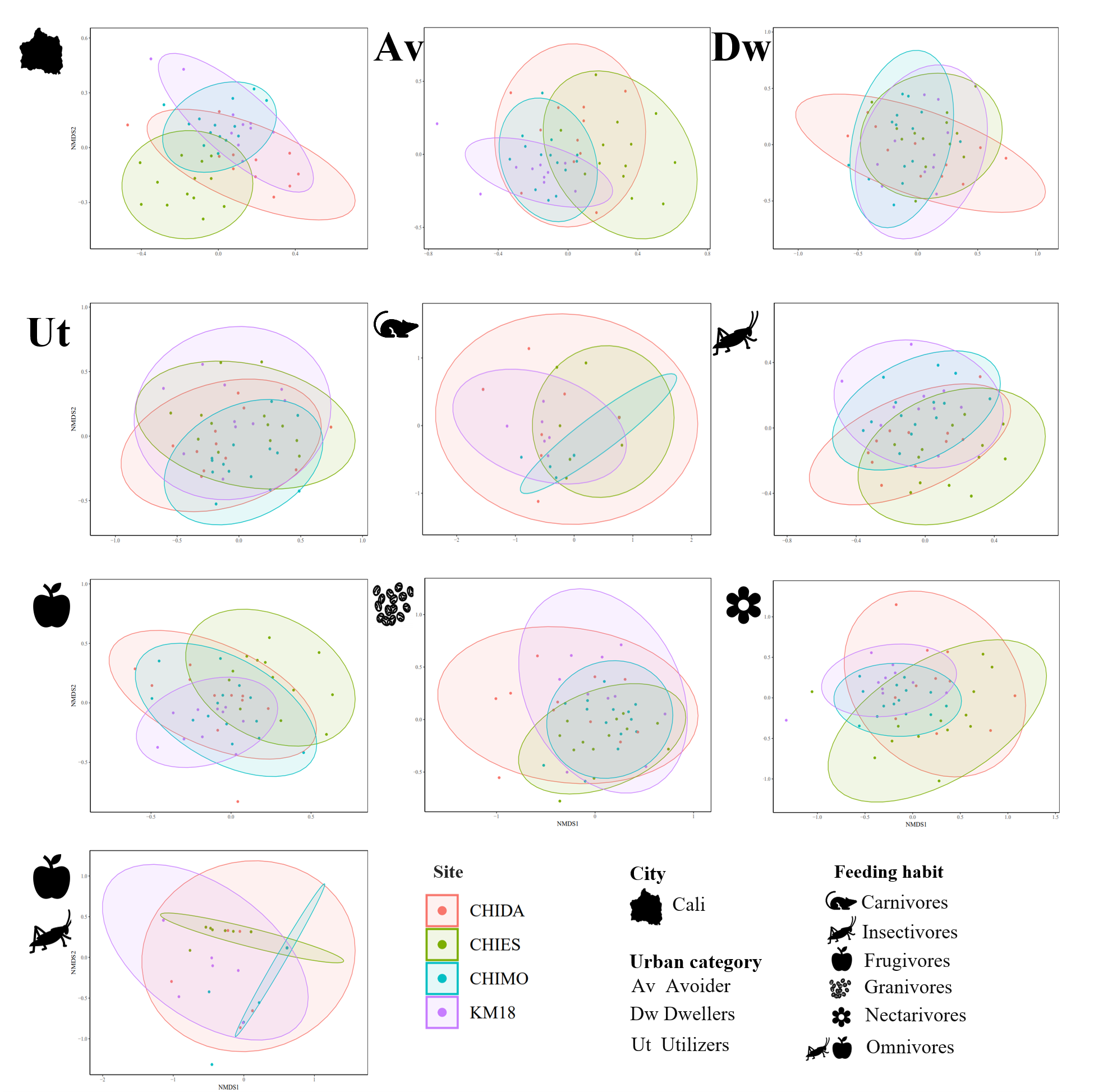

Supplement: S1 File — (DOCX) [file pone.0272754.s001.docx]
